# Arrhythmic risk stratification in psoriatic arthritis: a retrospective, electrogram-based comparative analysis

**DOI:** 10.1136/rmdopen-2026-007006

**Published:** 2026-06-24

**Authors:** Philipp Sewerin, Dimitrios Bismpos, Philipp Sebastian Lange, Neru Horstmann, Hendrik Sprave, Christian Ukena, Xenofon Baraliakos

**Affiliations:** 1 Rheumazentrum Ruhrgebiet Herne, Ruhr-University Bochum, Bochum, Germany; 2 Department of Cardiology/Angiology, Ruhr University Bochum, Marien Hospital Herne, Herne, Germany

**Keywords:** Arthritis, Psoriatic, Cardiovascular Diseases, Arthritis

## Abstract

**Background:**

Psoriatic arthritis (PsA) is a chronic inflammatory systemic disease that is associated with cardiovascular comorbidities, particularly with an increased risk of arrhythmias. Despite the evidence, a structured population-based risk stratification that relates ECG parameters to clinical characteristics in PsA is still lacking.

**Methods:**

Data from patients diagnosed with PsA who presented to our centre between 2014 and 2022 as well as from an age-matched and sex-matched control group of cardiovascular patients were collected. A variety of established ECG parameters associated with atrial and ventricular repolarisation and atrioventricular conduction as well as a comprehensive set of clinical variables were evaluated.

**Results:**

A total of 725 patients with PsA alongside 725 matched cardiovascular patients were enrolled (mean age 52.5±13.4 years). The median duration of PsA was 2 years. Patients with PsA had similar rates of first-degree atrioventricular block (AVB I°) with the control group (9.5% vs 10.2%; p=0.725). After excluding patients with known atrial arrhythmias, patients with PsA had higher rates of significant P-wave terminal force in V1 versus controls (30.2% vs 14.1%; p<0.001). Furthermore, patients with PsA had a longer corrected Tpeak-Tend interval (0.26±0.04 vs 0.23±0.05; p<0.001). After adjusting for age, among patients with PsA, male sex (16.4% vs 7.1%; p=0.006) as well as long disease duration (18.0% vs 6.9%; p<0.001) were associated with AVB I°.

**Conclusion:**

Patients with PsA are at a significantly increased risk for ECG abnormalities related to both atrial and ventricular repolarisation as well as atrioventricular conduction. Non-invasive, ECG-based tools for structured risk stratification could facilitate the early detection of patients at risk.

**WHAT IS ALREADY KNOWN ON THIS TOPIC**

- Psoriatic arthritis (PsA) is associated with an increased cardiovascular risk, particularly coronary artery disease; however, comparatively little is known regarding the risk of arrhythmia as well as its clinical predisposing factors.

**WHAT THIS STUDY ADDS**

- Patients with PsA demonstrated similar rates of first-degree atrioventricular block with cardiovascular controls, despite the perceived absence of typical risk factors.
- Furthermore, signs of impaired atrial and ventricular conduction were observed, especially in patients with long disease duration as well as presence of traditional cardiovascular risk factors.

**HOW THIS STUDY MIGHT AFFECT RESEARCH, PRACTICE OR POLICY**

- Chronic systemic inflammation in PsA may predispose to the development of an arrhythmogenic substrate, with early ECG-based stratification offering a potential approach to identify patients at increased risk of cardiac arrhythmias.

## Introduction

 Psoriatic arthritis (PsA) is one of the most common inflammatory rheumatic diseases and is closely associated with psoriasis. In Germany, approximately 0.5% of the population is affected, with an incidence of 83/100 000 person-years. The introduction of modern therapies has enabled the effective management of PsA in a significant proportion of patients, with the achievement of remission being recognised as the prevailing therapeutic target. In accordance with current guidelines, treatment decisions should not be based solely on peripheral arthritis but should also take into account other disease domains, including nail involvement, enthesitis and axial involvement. In addition to musculoskeletal manifestations, the focus of treatment is increasingly extending to comorbidities. Cardiovascular comorbidities are of particular significance in this context. This is partly due to the higher prevalence of cardiovascular comorbidities in patients with PsA and to the fact that achieving deep and sustained remission may lead to meaningful improvement in musculoskeletal manifestations and associated cardiometabolic morbidity.^1^

It has been well established that PsA is associated with an increased risk of cardiovascular disease, largely due to accelerated atherosclerosis mediated by chronic inflammation.^2^ In contrast to traditional cardiovascular comorbidities such as coronary artery disease (CAD) or stroke, comparatively little is known about the prevalence and progression of arrhythmias in patients with PsA. Previous studies have suggested that PsA may be associated with an increased incidence of arrhythmias,^3^ particularly atrial fibrillation (AF) and atrioventricular block (AVB).^4^ Chronic systemic inflammation is hypothesised to induce structural and electrophysiological changes in the myocardium, thereby promoting arrhythmogenesis.^5 6^ Indeed, inflammatory cytokines such as interleukin-1, interleukin-6, C reactive protein and tumour necrosis factor-alpha contribute to atrial electrical remodelling through multiple mechanisms, including gap junction dysfunction and disruption of intracellular calcium handling. These processes result in conduction slowing and increased ectopic activity, ultimately facilitating the development of AF.^7^,^9^

The aim of the study was to address the current gap in structured ECG-based arrhythmic risk stratification in PsA by evaluating ECG alterations compared with an age-matched and gender-matched cohort.

## Methods

### Study subjects and design

This retrospective analysis included patients with symptomatic PsA undergoing treatment during hospitalisation at our institution’s rheumatology clinic between 2014 and 2022. An age-matched and gender-matched group of cardiovascular patients without systemic inflammatory disease served as the control group. A variety of clinical parameters as well as ECG variables were recorded and analysed.

### Clinical and electrocardiographic assessment

As per protocol, all patients with PsA undergoing inpatient treatment received an extensive evaluation, including patient history of PsA, physical examination, assessment of medical comorbidities and risk factors, documentation of medications as well as a 12-lead ECG recording. The same evaluation was applied in an age-matched and gender-matched group of cardiovascular patients. Cardiovascular controls were included in the analysis once a systemic inflammatory disease was previously ruled out. In patients with PsA, a variety of disease-specific clinical and paraclinical parameters were collected and analysed when available: disease duration, the presence of the human leucocyte antigen B27 (HLA-B27) antigen and the type of immunomodulatory therapy. Age-matching and gender-matching aimed to assess the prevalence of ECG abnormalities in the PsA population relative to a comparable cardiovascular cohort, while retaining the meaningful between-group differences.

All patients included in the analysis received a 12-lead ECG recording. A variety of established ECG parameters related to atrial and ventricular depolarisation and repolarisation as well as atrioventricular conduction were evaluated. The ECG analysis was performed by three independent physicians (DB, HS, NH) who were blinded to the patients’ identity and data. Of note, the control group served the validation of the predictive value of the recorded ECG parameters. The ECG parameters included: P-wave amplitude (PWa), P-wave dispersion (PWd), P-wave peak time (PWPT), P-wave terminal force in V1 (PWTF), interatrial block (IAB), first-degree AVB (AVB I°), second-degree AVB (AVB II°), right bundle branch block (RBBB), left anterior fascicular block, left posterior fascicular block, left bundle branch block (LBBB), bifascicular block, early repolarisation, fragmented QRS complex, QT interval, corrected QT interval with Bazett formula (QTc), QT dispersion (QTd), Tpeak-Tend interval, corrected Tpeak-Tend interval (cTpTe) as well as Sokolow-Lyon index for left ventricular hypertrophy (Sokolow-Lyon Index for LVH). The evaluation of the ECG parameters was performed according to the established standards of clinical practice and, where appropriate, findings were reported with their rate of significance.^10^,^16^

### End points

The aim of this analysis was to assess the prevalence of ECG abnormalities in patients with diagnosed PsA, compared with a group of cardiovascular controls. In addition, we sought to explore possible associations between ECG abnormalities and clinical features of the disease, in order to identify patients at risk.

### Statistical analysis

Data are presented as mean±SD or number (percentage) unless otherwise specified. Continuous variables were tested for normal distribution with the Kolmogorov-Smirnov test and by visual inspection of quantile plots. Continuous variables between two groups were compared with the Wilcoxon rank-sum test, while the Kruskal-Wallis test was used for comparing continuous variables between more than two groups. Comparisons for categorical variables were performed with the Pearson’s χ^2^ test. To compare categorical variables between more than two groups, results were adjusted using the Bonferroni correction. Logistic regression analysis was performed to evaluate associations between ECG abnormalities and clinical features. Model fit was assessed using the Hosmer-Lemeshow test. A two-tailed p value of <0.05 was regarded as statistically significant. All statistical analyses were performed with SPSS statistical software (V.29.0, SPSS, Chicago, Illinois, USA). All authors had full access to the data and take full responsibility for their integrity.

## Results

### Patient characteristics

A total of 725 patients with PsA (mean age 52.5±13.4 years, 61.8% female sex) and available ECG data were included in our analysis, along with 725 cardiovascular controls without systemic inflammatory disease. The median duration of PsA was 2 years (IQR 0–8), while 21.6% of patients had a long disease duration (≥10 years). The HLA-B27 antigen was positive in 21.8% of patients (n=62/285). A total of 58.4% of patients were treated with disease-modifying antirheumatic drugs (DMARDs), most commonly conventional synthetic DMARDs (csDMARDs, 44.7%), while 58.7% used non-steroidal anti-inflammatory drugs (NSAIDs) and 34.7% received glucocorticoids. The prevalence of cardiovascular comorbidities among the patients with PsA as well as the rate of related prescribed medications are presented in table 1.

**Table 1:** Patient characteristics

Characteristics	Patients with PsA (n=725)	Cardiovascular control group (n=725)	P value
Age (years)	52.5±13.4	52.5±13.4	0.978
Female sex (%)	61.8	61.8	0.914
Median duration of PsA (years)	2 (0–8)	n.a.	n.a.
Long disease duration	21.6%	n.a.	n.a.
HLA-B27 antigen (%)	21.8	n.a.	n.a.
Arterial hypertension (%)	37.8	61.0	<0.001
Diabetes mellitus (%)	15.3	13.4	0.294
Hyperlipidaemia (%)	24.1	52.6	<0.001
Chronic kidney disease (%)	3.7	9.4	<0.001
Coronary artery disease (%)	4.7	29.2	<0.001
HFrEF (%)	0	6.1	<0.001
Atrial fibrillation (%)	1.5	17.1	<0.001
NSAIDs (%)	58.7	32.4	<0.001
DMARDs (%)	58.4	0.3	<0.001
csDMARDs (%)	44.7	0.3	<0.001
tsDMARDs (%)	1.7	0	<0.001
bDMARDs (%)	23.5	0	<0.001
Glucocorticoids (%)	34.7	6.6	<0.001
Antidepressants (%)	19.2	6.7	<0.001
Beta-blocker (%)	19.0	52.6	<0.001
MRA (%)	1.5	11.4	<0.001
SGLT2i (%)	3.0	21.8	<0.001
ACEi/ARB/ARNI (%)	38.5	55.3	0.004
Oral anticoagulation (%)	3.6	24.3	<0.001
Antiarrhythmic drugs (class I or III) (%)	0.3	5.7	0.002

ACEi, ACE inhibitors; ARB, angiotensin receptor blocker; ARNI, angiotensin receptor-neprilysin inhibitors; bDMARD, biologic DMARD; csDMARD, conventional synthetic DMARD; DMARD, disease-modifying antirheumatic drug; HFrEF, heart failure with reduced ejection fraction; HLA-B27, human leucocyte antigen B27; MRA, mineralocorticoid receptor antagonist; n.a., not available; NSAID, non-steroidal anti-inflammatory drug; PsA, psoriatic arthritis; SGLT2i, sodium-glucose co-transporter 2 inhibitors; tsDMARD, targeted synthetic DMARD.

The most prevalent clinical conditions in the age-matched and gender-matched group of cardiovascular patients included arterial hypertension (61.0%), CAD (29.2%) and AF (17.1%). The rate of prescribed anti-inflammatory drugs was very low, with the exception of NSAIDs (32.4%), whereas the use of cardiovascular drugs was consistent with the frequency of the respective diseases (table 1).

### Validation of ECG parameters

Among cardiovascular controls, patients with AF were older (59.6±11.6 vs 50.9±13.2 years; p<0.001) and presented more commonly hyperlipidaemia (HLP, 66.1% vs 49.7%; p<0.001) as well as chronic kidney disease (CKD, 23.4% vs 6.5%, p<0.001). Patients with AF had significantly higher rates of AVB I° (18.5% vs 8.5%; p<0.001) and IAB (6.5% vs 1.9%; p=0.018), as well as larger PWTF (4.98±2.83 vs 2.64±1.59 μVms; p<0.001). However, PWPT (53.7±15.9 vs 54.7±14.1 ms; p=0.157), PWa (0.13±0.09 vs 0.14±0.05 mV; p=0.356) and PWd (15.7±8.6 vs 15.5±10.1 ms; p=0.986) were similar between the two groups (online supplemental table S1).

Furthermore, patients with heart failure with reduced ejection fraction among cardiovascular controls were older (57.5±12.8 vs 52.1±13.3 years; p=0.009) and less often female (47.7% vs 62.5%; p=0.038). Furthermore, CKD (25.0% vs 8.2%; p<0.001) and CAD (50.0% vs 27.8%; p=0.003) were significantly more common in this population of patients. In addition, they presented higher rates of AVB I° (20.5% vs 9.4%; p=0.034) and of LBBB (15.9% vs 1.9%; p<0.001), as well as a longer QTc (465.3±42.4 vs 436.1±36.7 ms; p<0.0001) and numerically higher rates of significant cTpTe (15.9% vs 9.8%; p=0.199) (online supplemental table S2).

### ECG characteristics of patients with PsA

Compared with cardiovascular controls, patients with PsA had similar rates of significant PWPT (44.2% vs 46.8%; p=0.114). Furthermore, they presented higher rates of significantly low PWa (15.3% vs 7.4%; p<0.001) as well as higher rates of significant PWTF (30.4% vs 24.9%; p=0.022). Moreover, patients with PsA presented similar rates of AVB I° (9.5% vs 10.2%; p=0.725) with the cardiovascular controls (table 2A). After excluding patients with known atrial arrhythmias, patients with PsA still had a larger PWTF (3.03±2.29 vs 2.64±1.59 μVms; p<0.001), shorter PWa (0.10±0.04 vs 0.14±0.05; p<0.001) as well as similar rates of AVB I° (9.1% vs 8.4%; p=0.770) and statistically similar rates of IAB (8.4% vs 19.9%; p=0.237) compared with cardiovascular controls (table 2B).

**Table 2:** (A) ECG parameters associated with atrial conduction in patients with PsA, with known atrial arrhythmias and (B) ECG parameters associated with atrial conduction in patients with PsA, without known atrial arrhythmias

ECG parameters	Patients with PsA (n=725)	Cardiovascular control group (n=725)	P value
(A)			
PWPT (ms)	50.9±13.1	54.6±14.4	<0.001
Significant PWPT (%)	44.2	46.8	0.114
PWa (mV)	0.10±0.04	0.13±0.06	<0.001
Significant PWa (%)	15.3	7.4	<0.001
PWTF (μVms)	3.03±2.29	3.04±2.06	0.915
Significant PWTF (%)	30.4	24.9	0.022
PWd (ms)	13.3±13.2	15.5±9.8	<0.001
AVB I° (%)	9.5	10.2	0.725
IAB (%)	9.6	27.6	0.073
(B)			
PWPT (ms)	50.8±13.1	54.7±14.1	<0.001
Significant PWPT (%)	44.1	49.2	0.067
PWa (mV)	0.10±0.04	0.14±0.05	<0.001
Significant PWa (%)	15.4	6.7	<0.001
PWTF (μVms)	3.03±2.29	2.64±1.59	<0.001
Significant PWTF (%)	30.2	14.1	<0.001
PWd (ms)	13.3±13.3	15.5±10.1	0.001
AVB I° (%)	9.1	8.4	0.770
IAB (%)	8.4	19.9	0.237

AVB I°, first-degree atrioventricular block; IAB, interatrial block; PWa, P-wave amplitude; PWd, P-wave duration; PWPT, P-wave peak time; PWTF, P-wave terminal force.

Patients with PsA had higher rates of a positive Sokolow-Lyon Index for LVH (4.4% vs 1.2%; p<0.001) compared with cardiovascular controls. Conversely, cardiovascular controls had higher rates of LBBB (2.8% vs 0.5%; p=0.001). Regarding ventricular repolarisation, QTc was similar between the PsA and the control group (436.8±29.5 vs 437.9±37.7; p=0.500). However, patients with PsA presented a significantly longer cTpTe (0.26±0.04 vs 0.23±0.05; p<0.001), including higher rates of significant cTpTe (20.7% vs 10.2%; p<0.001) (table 3). Even after excluding patients receiving antidepressants, cTpTe was longer in the PsA group (0.26±0.04 vs 0.23±0.04; p<0.001).

**Table 3:** ECG parameters associated with ventricular depolarisation/repolarisation in patients with PsA

ECG parameters	Patients with PsA (n=725)	Cardiovascular control group (n=725)	P value
LBBB (%)	0.5	2.8	0.001
RBBB (%)	3.2	3.6	0.772
Bifascicular block (%)	1.9	4.3	0.015
Fragmented QRS (%)	0.3	1.2	0.064
Sokolow-Lyon Index for LVH (%)	4.4	1.2	<0.001
Early repolarisation (%)	0.5	2.1	0.018
QTc (ms)	436.8±29.5	437.9±37.7	0.500
QTd (ms)	17.7±10.3	20.8±12.2	<0.001
cTpTe (ratio)	0.26±0.04	0.23±0.05	<0.001
Significant cTpTe (%)	20.7	10.2	<0.001

cTpTe, corrected Tpeak-Tend interval; LBBB, left bundle branch block; LVH, left ventricular hypertrophy; PsA, psoriatic arthritis; QTc, corrected QT interval with Bazett formula; QTd, QT dispersion; RBBB, right bundle branch block.

To assess the robustness of our findings despite baseline differences between the PsA group and the control group, a multivariable binary logistic regression analysis adjusted for clinically relevant covariates was performed for AVB I°, PWTF and cTpTe, yielding results consistent with the primary analysis (online supplemental tables S3-S5).

### Association of ECG abnormalities with clinical variables

Among patients with PsA, those with AVB I° were older (63.7±9.1 vs 51.3±13.3 years; p<0.001), while the prevalence of AVB I° was higher in patients with a longer disease duration (≥10 years, 18.0% vs 6.9%; p<0.001), male sex (16.4% vs 7.1%; p=0.006), arterial hypertension (12.8% vs 7.5%; p=0.062), CKD (22.2% vs 9.0%; p=0.035), AF (36.3% vs 9.1%; p=0.015) and CAD (23.5% vs 8.8%; p=0.011). Furthermore, patients with significant PWTF had higher rates of AVB I° (13.2% vs 7.9%; p=0.038). On the contrary, patients on NSAIDs had a numerically lower prevalence of AVB I° (8.5% vs 14.1%; p=0.120). In a binary logistic regression, a long disease duration was independently associated with AVB I° (OR 2.25, 95% CI 1.19 to 4.27; p=0.013), although older age (≥65 years) showed the strongest association (table 4).

**Table 4:** Binary logistic regression for the prediction of AVB I° in patients with PsA

	OR (95% CI)
Male sex	2.16 (1.16 to 4.01)
Age ≥60 years	3.85 (1.98 to 7.48)
PsA duration ≥10 years	2.25 (1.19 to 4.27)
Arterial hypertension	1.03 (0.55 to 1.93)
Chronic kidney disease	1.79 (0.57 to 5.60)
Coronary artery disease	1.12 (0.37 to 3.42)
Atrial fibrillation	2.84 (0.72 to 11.23)

AVB I°, first-degree atrioventricular block; PsA, psoriatic arthritis.

Furthermore, patients with significant PWTF were older (53.8±11.9 vs 51.9±14.0; p=0.086) and more commonly male (34.2% vs 28.1%; p=0.096), although these associations did not reach statistical significance. In addition, the prevalence of significant PWTF in PsA was higher in patients with arterial hypertension (36.6% vs 26.6%; p=0.015) and HLP (38.3% vs 27.9%; p=0.011). There was no significant association with disease duration or with the type of immunomodulatory therapy, although significant PWTF was numerically more common in patients receiving glucocorticoids (43.3% vs 35.4%; p=0.164).

Finally, the prevalence of a significant cTpTe was higher in younger patients (49.4±14.6 vs 53.3±12.9 years, p=0.002) with male sex (30.1% vs 14.9%; p<0.001) and signs of LVH (40.6% vs 19.7%; p=0.012) as well as in patients treated with csDMARDs (25.4% vs 16.6%; p=0.069). On the contrary, significant cTpTe was less prominent in patients with hypothyroidism (7.6 vs 23.2%; p<0.001).

The presence of the HLA-B27 antigen was not associated with any of the mentioned ECG abnormalities.

## Discussion

In this study, we investigated the prevalence of ECG abnormalities in a PsA population by directly comparing them with an age-matched cohort for the first time. Our study provides the following novel insights: first, patients with PsA exhibited similar rates of AVB I° with cardiovascular controls, despite the perceived absence of traditional cardiovascular risk factors. Furthermore, patients with PsA demonstrated higher rates of abnormal PWa, PWTF and cTpTe compared with cardiovascular controls, suggesting impaired atrial and ventricular action potential dynamics. Finally, ECG screening appears to be a useful tool for identifying patients with PsA at risk.

Numerous studies have already shown that patients with PsA have an increased risk for cardiovascular events. PsA can be associated with subclinical vascular inflammation and also with the significantly higher incidence of obesity, metabolic syndrome and related potential cardiovascular complications.^1^ In our cohort, patients with PsA from routine clinical practice underwent a dedicated examination of the ECGs. Notably, only a very small fraction of these patients had previously received cardiological treatment or reported any subjective cardiac symptoms.

Surprisingly, we found comparatively high rates of AVB I° in the PsA population, despite lacking typical risk factors associated with cardiovascular disease. In addition, these patients had significantly lower prescription rates of beta-blockers and antiarrhythmic drugs, which are typically associated with prolonged atrioventricular conduction. This is particularly significant, as AVB I° has been associated with worse cardiovascular prognosis, including higher rates of pacemaker implantation, heart failure and death.^17 18^ A recent study by Almansouri *et al* likewise reported an increased cumulative incidence of bradycardia in patients with PsA; however, in that study, no direct age-matched comparison cohort was included and the exact rate of AVB was not mentioned in detail.^19^

In addition, we evaluated ECG parameters that, although not routinely incorporated into standard clinical diagnostics, are well established in arrhythmia-focused electrocardiographic risk stratification. When examining the PWTF, significantly higher rates of PWTF were found in PsA compared with the control group. Especially after excluding patients with known AF, the rates of significant PWFT were significantly higher in the PsA group compared with the control group (30.2% vs 14.1%; p<0.001). Similarly, patients with PsA in our study had a shorter PWa (0.10±0.04 vs 0.14±0.05; p<0.001). This is of great importance in daily clinical practice, as large PWTF and low PWa are directly associated with the development of AF.^20^ Our findings are consistent with those of Almansouri *et al*, who reported a high cumulative incidence of atrial arrhythmias in a PsA population, although they did not specify the exact type of atrial arrhythmias observed.^19^ Early detection and treatment of AF is vital in order to improve outcomes and quality of life.^21^ Importantly, the above findings are closely inter-related, as the presence of AVB has been shown to predict the subsequent development of AF.^22^

Notably, patients with PsA in our cohort exhibited higher rates of a positive Sokolow-Lyon Index for LVH. Although this ECG parameter has traditionally been used as a screening tool for LVH, more recent evidence indicates that its correlation with true LVH is weak.^23^ Accordingly, dedicated echocardiographic studies are warranted to determine whether patients with PsA are indeed at increased risk for LVH.

When looking at the ECG parameters of ventricular conduction, comparatively longer cTpTe values were observed in the PsA cohort, even when excluding patients receiving antidepressants, which are known for their QT-prolongation. The cTpTe has emerged as an important marker of ventricular repolarisation, as it has been demonstrated that longer cTpTe is associated with ventricular tachycardia and sudden cardiac death and is directly linked to cardiovascular mortality in large meta-analyses.^24^ These results could therefore partially explain the high mortality rate of patients with PsA, who mostly die from cardiovascular events.^25^ Here, too, further studies are needed to better elucidate this association.

Among patients with PsA, long disease duration as well as more traditional cardiovascular risk factors like older age, male sex and arterial hypertension were associated with a variety of ECG changes. Chronic systemic inflammation in patients with higher disease activity, paired with traditional cardiovascular risk factors, could create an arrhythmogenic substrate, as manifested by changes in 12-lead ECG. The use of drugs like DMARDs, NSAIDs and glucocorticoids was, as expected, more common in the PsA group; however, it was not statistically associated with any ECG abnormalities. Further trials are needed to clarify the possible arrhythmogenic effect of immunomodulatory drugs in PsA.

All in all, patients with PsA presented high rates of AVB I°, signs of impaired atrial function related to AF and even indications of prolonged ventricular repolarisation. This observation is important, as the prevalence of typical cardiovascular risk factors was comparably less prominent in the PsA group. We therefore hypothesise that arrhythmogenesis in PsA could follow different paths compared with a typical cardiovascular population. These findings underscore the need for targeted cardiological care in this particular group and highlight the importance of implementing targeted ECG screening to enable early detection, risk stratification and timely intervention in patients with PsA.

### Limitations

Our explorative, monocentric analysis had some limitations. Evaluation of ECG data was performed blindly by multiple physicians and consensus review, in accordance with the established clinical practice; however, the ECG interpretation can be subjective. Artificial intelligence-based systems could optimise ECG assessment and even uncover hidden patterns. It should also be noted that, despite the reasoning mentioned above and the sensitivity analysis, the unevenly distributed comorbidities could be viewed as potential confounders when assessing the prevalence of ECG abnormalities. In addition, while disease duration was analysed, further data regarding disease severity were not systematically assessed. Finally, analysis was based on data collected at enrolment, without subsequent clinical or ECG follow-up, so potential effects of disease progression or medication changes on clinical outcomes could not be evaluated. Therefore, our findings should be interpreted with caution regarding the incidence of clinical arrhythmias in this population. Despite these limitations, the available data already yield important implications regarding arrhythmic risk in PsA, providing a valuable basis for future research.

## Conclusion

Patients with PsA demonstrated electrocardiographic abnormalities of both atrial and ventricular repolarisation as well as atrioventricular conduction, which may indicate a predisposition to conditions such as AF, advanced conduction disease or even ventricular tachycardia. Further trials are needed to better understand the arrhythmic substrate and its possible association with high disease activity. Non-invasive, ECG-based tools for structured risk stratification could facilitate the early detection of patients at risk.

## Supplementary material

10.1136/rmdopen-2026-007006 online supplemental file 1

## Data Availability

Data are available on reasonable request.
