# Supplementary material for: Arrhythmic risk stratification in psoriatic arthritis: a retrospective, electrogram-based comparative analysis
Source: RMD Open. 2026 Jun 24;12(2):e007006. doi: 10.1136/rmdopen-2026-007006 (PMC13295788; doi:10.1136/rmdopen-2026-007006)
Supplement: online supplemental file 1 [file rmdopen-12-2-s001.docx]

**Supplement**

**Supplement Table S1 – Patient and ECG characteristics of cardiovascular patients with atrial fibrillation (AF)**

| Characteristics | Cardiovascular patients without AF  (n=601) | Cardiovascular  patients with AF  (n=124) | p |
| --- | --- | --- | --- |
| Age (years) | 50.9 ± 13.2 | 59.6 ± 11.6 | <0.001 |
| Female sex (%) | 61.7 | 61.3 | 0.927 |
| Arterial hypertension (%) | 59.4 | 68.5 | 0.126 |
| Diabetes Mellitus (%) | 13.6 | 12.1 | 0.645 |
| Hyperlipidemia (%) | 49.7 | 66.1 | <0.001 |
| Chronic kidney disease (%) | 6.5 | 23.4 | <0.001 |
| Coronary artery disease (%) | 29.8 | 26.6 | 0.480 |
| HFrEF (%) | 5.8 | 7.2 | 0.546 |
| Beta blocker (%) | 45.6 | 86.2 | <0.001 |
| MRA (%) | 8.8 | 24.2 | <0.001 |
| SGLT2i (%) | 19.1 | 34.7 | <0.001 |
| ACEi/ARB/ARNI (%) | 53.2 | 63.7 | 0.037 |
| Oral anticoagulation (%) | 10.8 | 89.5 | <0.001 |
| Antiarrhythmic drugs (class I or III) (%) | 2.9 | 18.5 | <0.001 |
| PWPT (ms) | 54.7 ± 14.1 | 53.7 ± 15.9 | 0.157 |
| Significant PWPT (%) | 49.3 | 45.2 | 0.407 |
| PWa (mV) | 0.14 ± 0.05 | 0.13 ± 0.09 | 0.356 |
| Significant PWa (%) | 47.6 | 40.3 | 0.139 |
| PWd (ms) | 15.5 ± 10.1 | 15.7 ± 8.6 | 0.986 |
| PWTF (μVms) | 2.64 ± 1.59 | 4.98 ± 2.83 | <0.001 |
| Significant PWTF (%) | 14.2 | 76.6 | <0.001 |
| AVB I° (%) | 8.5 | 18.5 | <0.001 |
| IAB (%) | 1.9 | 6.5 | <0.001 |

**Supplement Table S2 – Patient and ECG characteristics of cardiovascular patients with heart failure with reduced ejection fraction (HFrEF)**

| Characteristics | Cardiovascular patients without HFrEF  (n=680) | Cardiovascular  patients with HFrEF  (n=45) | p |
| --- | --- | --- | --- |
| Age (years) | 52.1 ± 13.3 | 57.5 ± 12.8 | 0.009 |
| Female sex (%) | 62.5 | 47.7 | 0.038 |
| Arterial hypertension (%) | 60.3 | 70.5 | 0.374 |
| Diabetes Mellitus (%) | 13.1 | 18.2 | 0.358 |
| Hyperlipidemia (%) | 51.5 | 68.2 | 0.042 |
| Chronic kidney disease (%) | 8.2 | 25.0 | <0.001 |
| Coronary artery disease (%) | 27.8 | 50.0 | 0.003 |
| Atrial fibrillation (%) | 16.9 | 20.5 | 0.537 |
| Beta blocker (%) | 49.8 | 95.4 | <0.001 |
| MRA (%) | 7.5 | 72.7 | <0.001 |
| SGLT2i (%) | 18.2 | 77.3 | <0.001 |
| ACEi/ARB/ARNI (%) | 52.9 | 86.4 | <0.001 |
| Oral anticoagulation (%) | 22.8 | 47.7 | <0.001 |
| Antiarrhythmic drugs (class I or III) (%) | 4.7 | 20.5 | <0.001 |
| AVB I° (%) | 9.4 | 20.5 | 0.034 |
| LBBB (%) | 1.9 | 15.9 | <0.001 |
| RBBB (%) | 3.1 | 11.4 | 0.017 |
| Bifascicular block (%) | 3.4 | 18.2 | <0.001 |
| Fragmented QRS (%) | 0.9 | 6.8 | 0.014 |
| Sokolow-Lyon Index for LVH (%) | 1.3 | 0 | 0.999 |
| Early repolarization (%) | 0 | 0.2 | 0.999 |
| QTc (ms) | 436.1 ± 36.7 | 465.3 ± 42.4 | <0.001 |
| QTd (ms) | 20.7 ± 12.1 | 22.9 ± 14.6 | 0.232 |
| cTpTe (ratio) | 0.23 ± 0.05 | 0.22 ± 0.02 | 0.392 |
| Significant cTpTe (%) | 9.8 | 15.9 | 0.199 |

**Supplement Table S3 – Binary logistic regression for the prediction of AVB I° in the whole population**

|  | OR (95% CI) |
| --- | --- |
| Psoriasis Arthritis | 1.25 [0.82 – 1.90] |
| Age ≥ 60 years | 3.73 [2.50 – 5.56] |
| Male sex | 2.20 [1.51 – 3.19] |
| Arterial hypertension | 1.23 [0.82 – 1.86] |
| Diabetes Mellitus | 0.80 [0.49 – 1.31] |
| Chronic kidney disease | 1.36 [0.76 – 2.43] |
| Coronary artery disease | 1.57 [0.98 – 2.49] |
| Atrial fibrillation | 1.19 [0.93 – 1.51] |

**Supplement Table S4 – Binary logistic regression for the prediction of significant P wave terminal force (PWTF) in the whole population**

|  | OR (95% CI) |
| --- | --- |
| Psoriasis Arthritis | 2.15 [1.62 – 2.87] |
| Age ≥ 60 years | 1.16 [0.87 – 1.55] |
| Male sex | 1.43 [1.11 – 1.85] |
| Arterial hypertension | 1.44 [1.09 – 1.88] |
| Diabetes Mellitus | 0.72 [0.49 – 1.05] |
| Chronic kidney disease | 1.43 [0.88 – 2.33] |
| Coronary artery disease | 0.35 [0.57 – 1.22] |
| Atrial fibrillation | 2.87 [2.29 – 3.59] |

**Supplement Table S5 – Binary logistic regression for the prediction of significant corrected Tpeak-Tend interval (cTpTe) in the whole population**

|  | OR (95% CI) |
| --- | --- |
| Psoriasis Arthritis | 2.24 [1.60 – 3.12] |
| Age ≥ 60 years | 0.86 [0.60 – 1.23] |
| Male sex | 2.08 [1.55 – 2.80] |
| Arterial hypertension | 1.13 [0.82 – 1.56] |
| Diabetes Mellitus | 0.82 [0.52 – 1.29] |
| Chronic kidney disease | 0.81 [0.40 – 1.64] |
| Coronary artery disease | 0.63 [0.37 – 1.05] |
| Heart failure with reduced ejection fraction | 1.83 [0.77 – 4.35] |
